# Study of the grafting compatibility of the apple rootstock 12–2, resistant to apple replant diseases (ARD)

**DOI:** 10.1186/s12870-022-03847-8

**Published:** 2022-09-30

**Authors:** Yunfei Mao, Xueli Cui, Haiyan Wang, Xin Qin, Yangbo Liu, Yanli Hu, Xuesen Chen, Zhiquan Mao, Xiang Shen

**Affiliations:** National Key Laboratory of Crop Biology, College of Horticulture Science and Engineering, Shandong Agricultural University, Tai’an, Shandong People’s Republic of China

**Keywords:** Resistant rootstock, Aboveground physiological parameters, Grafted combinations

## Abstract

**Background:**

Cultivation of resistant rootstocks can effectively prevent apple replant disease (ARD), and grafting tests are an important means of evaluating the compatibility of rootstocks with scions.

**Methods:**

The apple rootstocks 12–2 (self-named) and *Malus hupehensis* Rehd. (PYTC) were planted in a replanted 20-year-old apple orchard. The two rootstocks were grafted with scions of 13 apple varieties. Multiple aboveground physiological parameters of the grafted combinations were measured and evaluated to verify the grafting affinity of 12–2 with the scions as compared to *Malus hupehensis* Rehd. (PYTC).

**Results:**

The graft survival rate and graft interface healing of 12–2 did not differ significantly from those of PYTC. Mechanical strength tests of the grafted interfaces showed that some mechanical strength indices of Redchief, Jonagold, Starking, Goldspur and Yinv apple varieties were significantly higher when they were grafted onto 12–2 compared to the PYTC control. The height and diameter of shoots and the relative chlorophyll content, photosynthetic and fluorescence parameters, antioxidant enzyme activities and malondialdehyde content of leaves showed that Fuji 2001, Tengmu No.1, RedChief, Gala, USA8, and Shoufu1 grew similarly on the two rootstocks, but Tianhong 2, Lvguang, Jonagold, Starking, Goldspur, Yinv and Luli grew better when grafted onto 12–2 than onto the PYTC control. The rootstock 12-2, therefore, showed good grafting affinity.

**Conclusion:**

These results provide experimental materials and theoretical guidance for the cultivation of a new grafting compatible rootstock to the 13 studied apple cultivars.

## Introduction

Apple is a popular fruit that is cultivated worldwide (except tropical countries). In recent years, the output and cultivated area of apple have increased steadily, playing an important role in the development of the rural economy and increasing farmers' incomes in China [32]. Apples rely mainly on grafting for propagation. Above the graft interface is the scion and below it is the rootstock. The rootstock is an important component of fruit trees: it not only absorbs soil moisture and stores and transport to the scion some nutrients, but also converts inorganic soil nutrients into organic substances for plant utilization, thereby strongly affecting the growth and development of fruit trees [34].

Apple replant disease (ARD) occurs widely in old orchards around the world, severely limiting the healthy development of the apple industry [18, 21]. The causes of ARD are complex. *Pythium, Phytophthora* and other fungi such as *Ilyonectria*, *Rhizoctonia* and others are considered to be the main causes of the disease [49]. The development of improved ARD-resistant rootstocks is a long-term effective measure for the prevention and control of ARD [16]. The Geneva rootstocks G.11, G.16, and G.41 are reportedly tolerant to some of the causative agents implicated in ARD and they have been promoted in some parts of Europe and the United States [25]. However, the causal pathogens may vary across different geographic regions [47], and these Geneva rootstocks have not been promoted in China for various reasons.

Grafting apples onto rootstocks with low ARD resistance has the disadvantages of shorter lifespans and shallower root systems [39]. The cultivation of highly resistant rootstocks can effectively control pests and diseases in the soil, alleviating ARD caused by some pathogens [26], enhancing plant stress resistance and increasing fruit yield and quality [48]. *Malus hupehensis* Rehd. (PYTC) is recognized as a rootstock with strong resistance to ARD in China [42]. It exhibits good grafting affinity with various apple varieties, apomixis traits, some dwarfing effect and it is easy to propagate by cuttage [44]. However, when PYTC is used as a rootstock for 'Fuji' apple scions, the resulting tree is too large, the fruiting rate is low, the fruit are small, the salt and drought resistance are poor and the survival rate in northwest China is low [14]. Grafting tests are an important means for evaluating the compatibility between rootstocks and scions. Rootstocks are selected for rooting and grafting capacity, abiotic and biotic stress tolerance and their ability to beneficially alter scion phenotypes [41].

In the process of apple rootstock breeding, it is very important to evaluate grafting compatibility. A closer genetic relationship between a *Malus* rootstock and scion is not a guarantee of better grafting survival, and incompatible *Malus* species cannot be called apple rootstocks [1]. There are many methods for evaluating grafting compatibility of scion–rootstock combinations. At present, grafting compatibility is typically assessed using multiple indicators such as graft survival rate, plant growth, and physiological measurements of scion–rootstock combinations [36]. Graft survival rate is an important parameter for measuring grafting technology, and a higher survival rate is the basis of grafting feasibility [9]. Incompatibility between a scion and rootstock will affect their communication, impairing the transfer of water and nutrients between them and resulting in the death of the scion and the failure of the graft [11].

Therefore, breeding ARD-resistant apple rootstock varieties with completely independent intellectual property rights, using China's unique apple rootstock resources and evaluating their grafting compatibility, are important for promoting the resistance breeding of apple rootstocks in China. Our research groups used the patented technology of *in situ* breeding [29] to select a new elite apple rootstock line named 12–2 that is tolerant to ARD. It is a new line of *Malus* species that has not been identified previously. In our previous research, ARD had significant effects on several parameters of M.9T337 and M.26 rootstocks, but had no significant effect on 12–2 [16, 17]. On this basis, we used 12–2 and PYTC as control, planted in an ARD orchard as experimental rootstock materials and grafted them with 13 apple varieties, including Fuji 2001 and Tianhong 2. We evaluated the grafting compatibility of 12–2 using PYTC as the control to provide a theoretical basis for the subsequent popularization of rootstock 12–2.

## Materials and methods

### Plant materials and experimental plots

The experiment was carried out from March 2016 to March 2019 in an ARD experimental orchard, originally planted with 20-year-old Red Fuji on *Malus* × *robusta* (CarriŠre) Rehder, at the National Key Seedling Breeding Base of Shandong Agricultural University, Tai'an, Shandong, China. The soil texture was a brown loam. The soil bulk density was 1.2 g·cm^−3^, and its pH was 5.3. The soil nutrient contents included 5.9 mg·kg^−1^ ammonium nitrate, 8.4 mg·kg^−1^ nitrate nitrogen, 103.7 mg·kg^−1^ available phosphorus, 18.3 mg·kg^−1^ available potassium and 12.1 g·kg^−1^ organic matter. The test material was the 12–2, an ARD-tolerant rootstock, selected through our patented in situ breeding technology, and PYTC (*Malus hupehensis* Rehd.) seedlings purchased from Shandong Horticultural Techniques & Services Co. Ltd., Tai’an, Shandong, China, that served as control. In early November 2017, a storage trench was dug in the cool shade at the research base. The trench was 80 cm deep, 100 cm wide and 100 cm long. During November 2017, scions of 13 apple varieties were obtained from the Shandong Institute of Pomology, Tai'an, Shandong, China; Shandong Horticultural Techniques & Services Co. Ltd., Tai'an, Shandong, China; and the National Research Center for Apple Engineering and Technology, Tai'an, Shandong, China. The scion varieties included: Fuji 2001 (2001), Tianhong 2 (TH2H), Lvguang (LG), Tengmu No.1 (TMYH), Redchief (SH), Gala (GL), Jonagold (QNJ), USA8 (MB), Starking (HX), Goldspur (JAS), Yinv (YN), Luli (LL) and Shoufu1 (SF1H). We placed 2–3 cm of clean sand (water content ≤ 10%) in the trench, tied the scion strips with classification marks and placed them obliquely in the trench. We then filled the trench completely with sand and covered it with rain-proof materials.

### Experimental treatments

Beginning in early March 2016, 12–2 tissue cultured seedlings were subcultured using the methods of Mao et al. [16, 17], under the same conditions for 8 months. The medium was based on 1 L MS medium containing 30 g L^−1^ sucrose (Sigma-Aldrich Co. Ltd., Shanghai, China), 7.5 g L^−1^ agar (Sigma-Aldrich), 0.6 mg L^−1^ 6-BA (Sigma-Aldrich) and 0.2 mg L^−1^ IBA (Sigma-Aldrich) at pH 5.8. Tissue culture was performed as described in Mao et al. [17]. In early January 2017, 12–2 tissue cultured seedlings from multiple subcultures were inoculated into rooting medium based on 1 L 1/2 MS medium and containing 20 g L^−1^ sucrose, 7.5 g L^−1^ agar, 0.2 mg L^−1^ 6-BA, and 1.0 mg L^−1^ IBA at pH 5.8. Five buds were inoculated into each vial of induction medium and placed in a tissue culture chamber at 25 ± 2 ℃ with a 10 h light period and an illumination intensity of 1000 lx. In early March 2017, rooted seedlings with consistent growth and with four to five leaves, were selected and transplanted into sterile substrate after hardening off. In early February 2017, purchased PYTC seeds were layered at 4 °C for 30 d; after the seeds had turned white, they were sown in sterile medium.

When the 12–2 and PYTC seedlings had both grown six to seven leaves, they were planted in the ARD test field at a row spacing of 1.5 m × 2 m in early April 2017; there were 300 plants of each variety. In early March 2018, 13 apple varieties were grafted onto the two-year-old rootstocks at a height of 8 cm from the ground, with 20 plants of each scion variety. Normal water and fertilizer management were performed throughout the experiment.

The graft survival rate and graft interface healing were recorded in early April 2018. The height and diameter of shoots, and the relative chlorophyll content, photosynthetic and fluorescence parameters, antioxidant enzyme activities and malondialdehyde (MDA) contents on leaves were measured every 30 days for three consecutive months, beginning in early July 2018. Leaf measurements were performed on the fifth to the seventh uninjured, fully expanded-adult leaves of each plant (measured from the bottom up). The mechanical strength of the grafted interface was measured in mid-March 2019, during the dormant period. Fifteen grafted seedlings were randomly selected for each scion–rootstock combination. Whole plants were planed out for mechanical strength testing, according to GB/T 1927–2009 [7]. There were three biological replicates for each treatment.

### Experimental methods

#### Graft survival rate and grafted interface healing

After grafting, the buds were wiped every 5 days (5 times in total).

$$Graft\;survival\;rate\;(\%)\;=\;number\;of\;surviving\;scions/number\;of\;grafted\;scions\;\times\;100.$$

Grafted interface healing was assessed by visual inspection.

#### Mechanical strength test of the grafted interface

The grafted interface of each grafted seedling was sawn. Based on GB/T 1929–2009 [6] and Wang et al. [38], with slight modifications, a cylindrical specimen was obtained according to the specifications in Table 1.

**Table 1:** The specifications of cut specimens required for different mechanical strength measurements of each grafted interface

**Mechanical strength** **measurement indicators**	**Total** number	**Average diameter** (mm)	**Average length** (mm)
Compressive elastic modulus (MPa)	78	13 ± 0.1	30 ± 2
Grain compressive strength (MPa)	78	13 ± 0.1	30 ± 2
Grain tensile strength (MPa)	78	13 ± 0.1	160 ± 2
Torsional strength (MPa)	78	13 ± 0.1	200 ± 2
Peak torque (N•m)	78	13 ± 0.1	200 ± 2

The test methods referred to GB/T 1928–2009 [5]. Instruments used in this test were a WDW-5E microcomputer-controlled electronic universal testing machine (Ji'nan Shijin Group Co. Ltd., Ji'nan, Shandong, China), a TNS-DW microcomputer-controlled torsion testing machine (Ji'nan Shijin Group Co. Ltd., Ji'nan, Shandong, China), a tape measure, a vernier caliper and a saw planer for woodworking.

#### Shoot height and shoot diameter

The shoot height was measured with a ruler, starting from the graft interface, and shoot diameter was measured 1 cm above the graft interface with vernier calipers.

#### Leaf relative chlorophyll content

The leaf relative chlorophyll content was measured with a SPAD-502 portable chlorophyll meter (Beijing Harvesting Science and Technology Co., Ltd., Beijing, China) [16].

#### Leaf photosynthetic parameters

The leaf net photosynthetic rate (*P*_n_), intercellular CO_2_ concentration (*C*_i_), stomatal conductance (*G*_s_), and transpiration rate (*T*_r_) were measured with a CIRAS-2 portable photosynthesis measurement system (PP-Systems, Hansha Scientific Instruments, Beijing, China) [16].

#### Leaf fluorescence parameters

The maximum potential quantum efficiency of PSII (*F*_v_/*F*_m_), actual photochemical efficiency of PSII (*Φ*_PSII_), non-photochemical quenching coefficient (NPQ), photochemical quenching coefficient (qP), and electron transfer rate (ETR) were measured with a German WALZ Junior-PAM portable fluorometer (Zealquest Scientific and Technology Co., Ltd., Shanghai, China) [16].

#### Leaf antioxidant enzyme activities and malondialdehyde (MDA) content

Superoxide dismutase (SOD) activity was measured by the method of Sun et al. [31], peroxidase (POD) activity by the guaiacol method described in Omran [22], and catalase (CAT) activity by the method of Singh et al. [30]. MDA content was determined by the thiobarbituric acid (TBA) method [15].

### Data analysis

Mean differences between treatments were compared using Student’s *t*-test and Duncan's multiple range test (DMRT) at the 0.05 probability level, unless otherwise stated.

Morphological indicators (survival rate, grafting interface healing, etc.) and physiological indicators (photosynthetic parameters, antioxidant enzyme activity, etc.) are mainly used to reflect grafting compatibility [36]. Because the physiological indicators were measured for three consecutive months, grafting compatibility could not be evaluated solely on the basis of a specific indicator in an individual month. Therefore, we performed principal component analysis on the physiological indicators. Principal component analysis was first performed on 13 scion–rootstock combinations using Origin 2021. The remaining scion–rootstock combinations that could not distinguish significant differences were analyzed by SPSS19.

SPSS was used to perform principal component analysis. First, the measured parameters for different scion–rootstock combinations were subjected to dimension reduction factor analysis. Then, descriptive analysis of the measured data was performed and the scores for six groups (12–2 in July, PYTC in July, 12–2 in August, PYTC in August, 12–2 in September, and PYTC in September) were calculated for each scion. And the score as the ratio of the eigenvalues corresponding to each principal component to the sum of the total eigenvalues of the extracted principal components. Finally, differences in growth vigor among rootstock and scion combinations in different months were obtained. In the equations below, *F*_*1*_*–F*_*i*_ represents the *1*–*i*^*th*^ principal components; *X*_*1*_–*X*_*16*_ correspond to the 16 measured parameters and the number before each *X* is the eigenvector corresponding to the test parameter divided by the square root of the eigenvalue, corresponding to the principal component; (*α*_*1(1)*_ represents the square root of the first eigenvalue of the first principal component; *α*_*1(i)*_ represents the square root of the first eigenvalue of the *i*-th principal component, and so forth).

$$F_1=\alpha_{1(1)}X_1+\alpha_{2(1)}X_2+\alpha_{3(1)}X_3+\alpha_{4(1)}X_4+\alpha_{5(1)}X_5+\alpha_{6(1)}X_6+\alpha_{7(1)}X_7+\alpha_{8(1)}X_8+\alpha_{9(1)}X_9+\alpha_{10(1)}X_{10}+\alpha_{11(1)}X_{11}+\alpha_{12(1)}X_{12}+\alpha_{13(1)}X_{13}+\alpha_{14(1)}X_{14}+\alpha_{15(1)}X_{15}+\alpha_{16(1)}X_{16\dots\dots}$$

$$F_i=\alpha_{1(i)}X_1+\alpha_{2(i)}X_2+\alpha_{3(i)}X_3+\alpha_{4(i)}X_4+\alpha_{5(i)}X_5+\alpha_{6(i)}X_6+\alpha_{7(i)}X_7+\alpha_{8(i)}X_8+\alpha_{9(i)}X_9+\alpha_{10(i)}X_{10}+\alpha_{11(i)}X_{11}+\alpha_{12(i)}X_{12}+\alpha_{13(i)}X_{13}+\alpha_{14(i)}X_{14}+\alpha_{15(i)}X_{15}+\alpha_{16(i)}X_{16.}$$

The principal component score was calculated as the ratio of the eigenvalues corresponding to each principal component to the sum of the total eigenvalues of the extracted principal components:

$$F=\lambda_1F_1+\lambda_2F_2+\lambda_3F_3+\dots\dots+\lambda_iF_i$$

where *λ*_*1*_–*λ*_*i*_ represent the contribution rates of the *1*–*i*^*th*^ principal components.

## Results

### Graft survival rate

The graft survival rate of apple scions grafted onto 12–2 was 95.77%, and that of scions grafted onto PYTC was 94.62%; there was no significant difference in graft survival rate between the two rootstocks (Table 2), showing that 12–2 has similar grafting compatibility than the control PYTC.

**Table 2:** Graft survival rates of different scion–rootstock combinations

Treatment	2001	TH2H	LG	TMYH	SH	GL	QNJ	MB	HX	JAS	YN	LL	SF1H	Total
12–2														
Number of grafted scions	20	20	20	20	20	20	20	20	20	20	20	20	20	260
Number of surviving scions	20	20	20	19	20	20	18	19	20	18	16	20	19	249
PYTC														
Number of grafted scions	20	20	20	20	20	20	20	20	20	20	20	20	20	260
Number of surviving scions	20	20	20	20	19	18	20	19	20	18	16	19	17	246

### Grafted interface healing

Based on the healing status of the grafted interface, the scion–rootstock combinations could be divided into three categories: ‘strongly compatible’, ‘semi-compatible’ and ‘incompatible’ [3]. ‘Strong compatibility’ means that the interface healed well without the phenomenon of ‘big and small feet’. ‘Semi-compatibility’ refers to slow healing of the graft wound, followed by deformity and swelling, resulting in the growth phenomenon of ‘big feet’ with ‘big on the top and small on the bottom’, or ‘big on the bottom and small on the top’. ‘Incompatibility’ refers to a lack of budding after grafting and scion necrosis. Field investigations of the morphology of the grafted interface showed that the scion–rootstock combinations exhibited either strong compatibility or semi-compatibility. The 14 strongly compatible combinations were Fuji 2001, Tianhong 2, Tengmu No.1, Redchief, Goldspur, Yinv, Luli, and Shoufu1, grafted onto 12–2; and Fuji 2001, Tianhong 2, Lvguang, Tengmu No.1, Redchief, and Shoufu1, grafted onto PYTC. The 8 semi-compatible combinations that were ‘big on the top and small on the bottom’ were Lvguang, Jonagold, USA8, and Starking, grafted onto 12–2; and Gala, Jonagold, Goldspur, and Luli, grafted onto PYTC. The 4 combinations that were ‘big on the bottom and small on the top’ were Gala grafted on 12-2; and USA8, Starking and Yinv, grafted onto PYTC. There were no incompatible combinations.

### Mechanical strength of the grafted interface

**Fig. 1:**
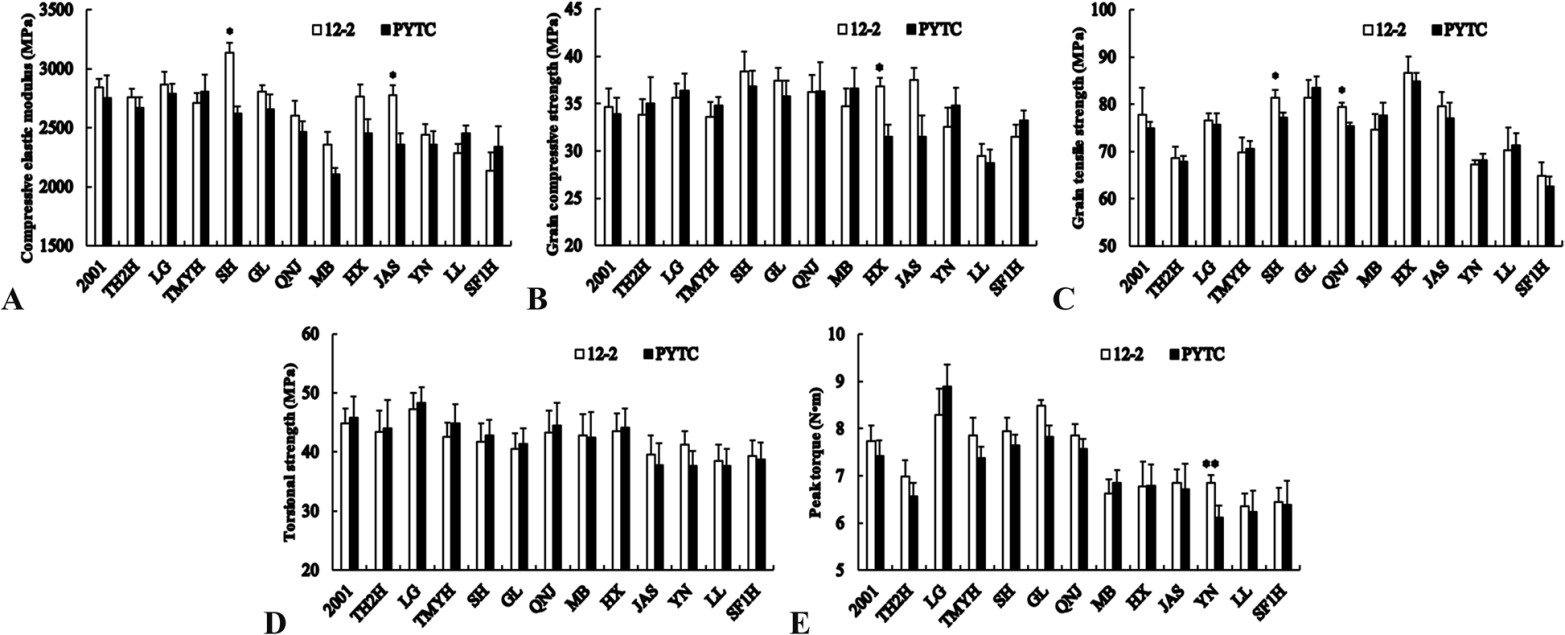
Mechanical strength of the grafted interface in different scion–rootstock combinations. **A** Compressive elastic modulus, **B** Grain compressive strength, **C** Grain tensile strength, **D** Torsional strength, and **E** Peak torque. Note: Student’s *t*-test was used to assess the significance of differences between the two rootstocks. *P < 0.05, **P < 0.01

Mechanical strength differed among the grafted interfaces of the different scion–rootstock combinations (Fig. 1). The compressive elastic modulus and grain tensile strength of Redchief, the grain tensile strength of Jonagold, the grain compressive strength of Starking, the compressive elastic modulus of Goldspur and the peak torque of Yinv, were significantly higher when these scions were grafted onto 12–2 rather than on the PYTC control. There were no significant differences in mechanical strength, indicators of any other scion–rootstock combinations. In general, these results showed that the mechanical strength of apple scions grafted onto 12–2 was not worse and was sometimes better than that of the same scions grafted onto PYTC.

### Shoot height

**Fig. 2:**
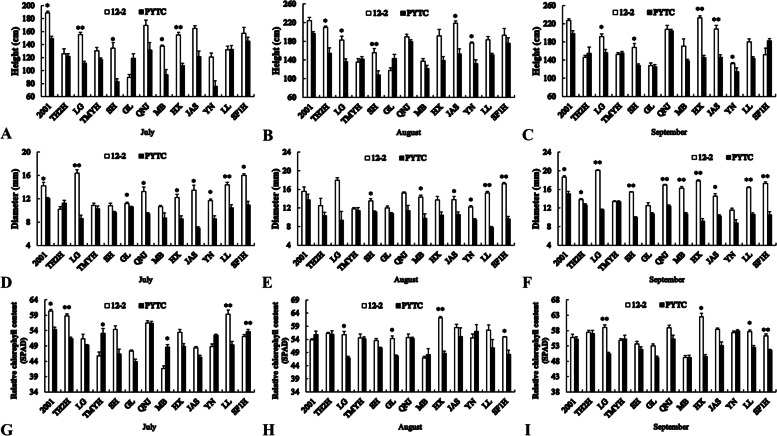
Growth and chlorophyll content of different scion–rootstock combinations. **A**–**C** Shoot height, **D**–**F** shoot diameter, and **G**–**I** relative chlorophyll content. *P < 0.05, **P < 0.01, Student’s *t*-test

In general, the shoots of scions grafted onto rootstock 12–2 were taller than those of the same scions grafted onto PYTC (Fig. 2A–C). The exceptions were Gala and Luli in July; Gala in August; and Tianhong 2, Tengmu No.1, Gala, Jonagold and Shoufu1 in September. In July, the heights of Fuji 2001, Lvguang, Redchief, USA8, and Starking were significantly greater on 12–2 than on PYTC; in August, the heights of Tianhong 2, Lvguang, Redchief, Goldspur and Yinv were significantly greater on 12–2 than on PYTC; and in September, the heights of Lvguang, Redchief, Starking, Goldspur and Yinv were significantly greater on 12–2 than on PYTC.

### Shoot diameter

Trends in shoot diameter were similar to those of shoot height, with the exceptions of Tianhong 2 in July and Tengmu No.1 in September (Fig. 2D–F). In July, the diameters of Fuji 2001, Lvguang, Gala, Jonagold, Starking, Goldspur, Yinv, Luli and Shoufu1 were significantly greater on 12–2 than on PYTC; in August, the diameters of Redchief, USA8, Goldspur, Yinv, Luli and Shoufu1 were significantly greater on 12–2 than on PYTC; and in September, the diameters of all scions except Tengmu No.1, Gala and Yinv were significantly greater on 12–2 than on PYTC. This also reinforce the indication that the rootstock 12–2 has better grafting compatibility than the control PYTC.

### Leaf relative chlorophyll content

Trends in leaf relative chlorophyll content differed from those of shoot height and shoot diameter (Fig. 2G–I). Chlorophyll contents of Fuji 2001 and Tianhong 2 (both on July), Lvguang (July and August), Gala (August), Starking (August and September), Luli (July and September), and Shoufu1 (August and September) were significantly higher on 12–2 than on PYTC. By contrast, chlorophyll contents of Tengmu No.1, USA8, and Shoufu1 (all on July), were significantly lower on 12–2 than on PYTC.

### Leaf photosynthetic parameters

**Fig. 3:**
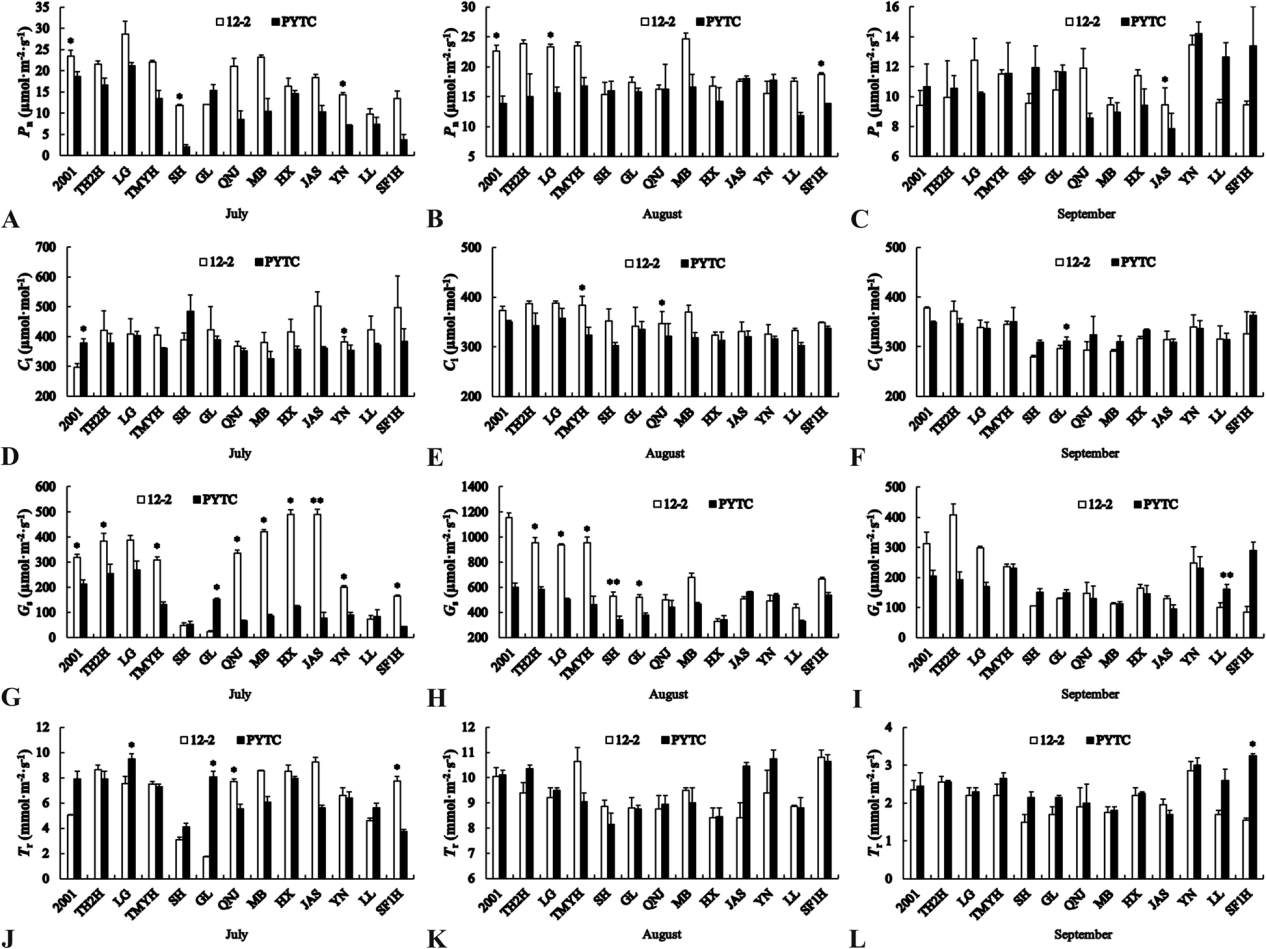
Leaf photosynthetic parameters of different scion–rootstock combinations. **A**–**C***P*_n_, **D**–**F***C*_i_, **G**–**I***G*_s_, **J**–**L***T*_r_. *P < 0.05, **P < 0.01, Student’s *t*-test

Leaf photosynthetic parameters differed among different scion–rootstock combinations (Fig. 3). The *P*_n_ of Fuji 2001 (July and August), Lvguang (August), Redchief (July), Goldspur (September), Yinv (July) and Shoufu1 (August) was significantly higher on 12–2 than on PYTC. The *C*_i_ of Tengmu No.1 (August), Jonagold (August) and Yinv (July) was significantly higher on 12–2 than on PYTC. By contrast, the *C*_i_ of Fuji 2001 (July) and Gala (September) was significantly lower on 12–2. The effect of rootstock on the *G*_s_ of Gala differed between months: *G*_s_ of Gala was significantly lower on 12–2 than on PYTC in July, but significantly higher on 12–2 in August. The *G*_s_ of most scion–rootstock combinations was significantly higher on 12–2 than on PYTC for one or more months from July to September. An exception was Luli grafted onto the rootstock 12–2, whose *G*_s_ in September was significantly lower than that of the control PYTC. The effect of rootstock on the *T*_r_ of Shoufu1 also differed among months: *T*_r_ of Shoufu1 was significantly higher on 12–2 than on PYTC in July, but significantly lower on 12–2 in September. In July, *T*_r_ of Jonagold was significantly higher on 12–2 than on PYTC and *T*_r_ of Lvguang and Gala was significantly lower on 12–2.

### Leaf fluorescence parameters

**Fig. 4:**
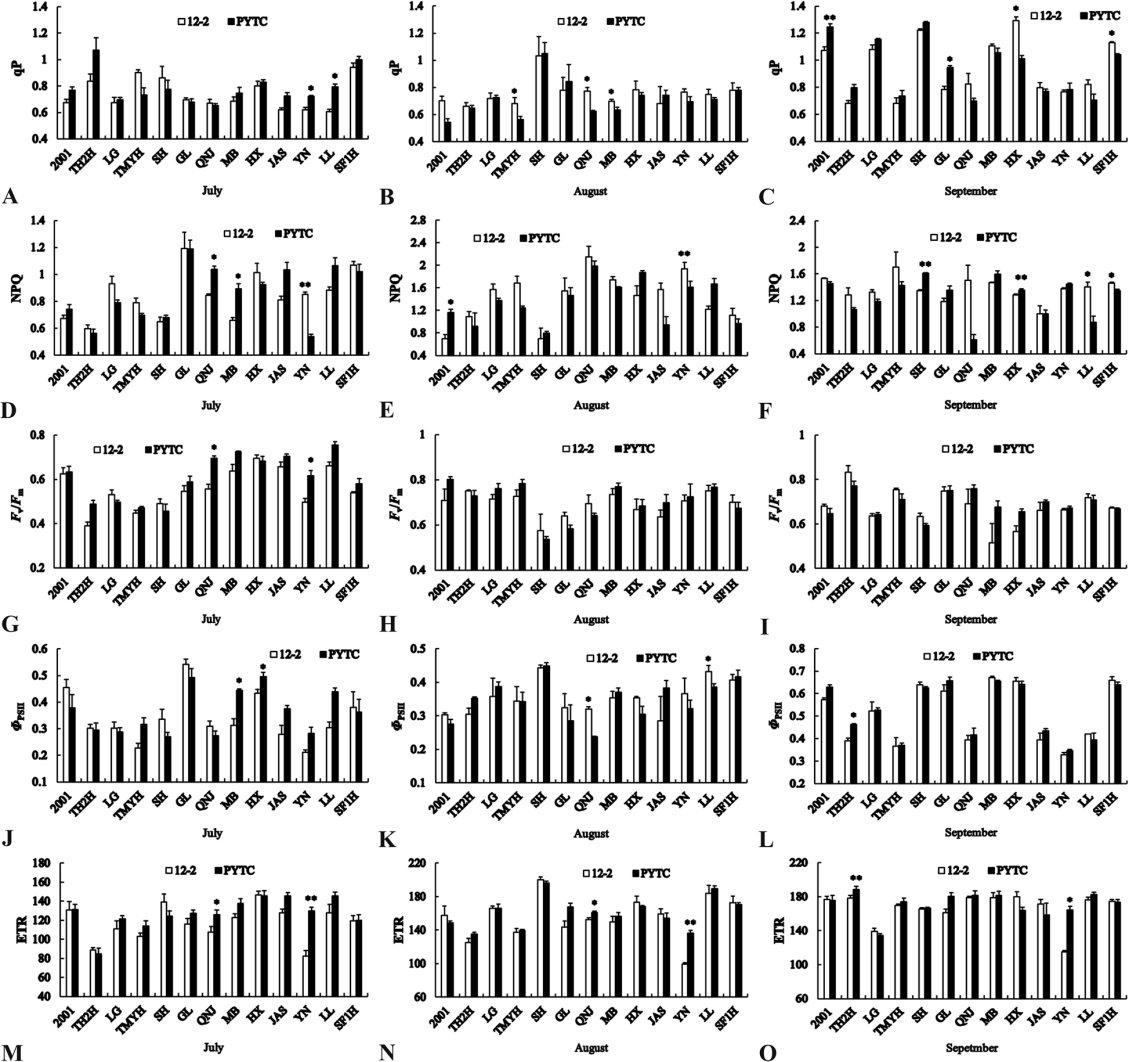
Leaf fluorescence parameters of different scion–rootstock combinations. **A**–**C** qP, **D**–**F** NPQ, **G**–**I***F*_v_*/F*_m_, **J**–**L***Φ*_PSII_, and **M**–**O** ETR. *P < 0.05, **P < 0.01, Student’s *t*-test

As shown in Fig. 4, some leaf fluorescence parameters of Jonagold (NPQ, *F*_v_/*F*_m_, and ETR), USA8 (NPQ and *Φ*_PSII_), Starking (*Φ*_PSII_), Yinv (qP, *F*_v_/*F*_m_, and ETR), and Luli (qP) were significantly lower on 12–2 than on PYTC in July, whereas the NPQ of Yinv was significantly higher on 12–2 than on PYTC. In August, some leaf fluorescence parameters of Tengmu No.1 (qP), Jonagold (qP and *Φ*_PSII_), USA8 (qP), Yinv (NPQ) and Luli (*Φ*_PSII_) were significantly higher on 12–2 than on PYTC, whereas the NPQ of Fuji 2001, the ETR of Jonagold and the ETR of Yinv were significantly lower on 12–2. In September, the qP of Fuji 2001, the *Φ*_PSII_ and ETR of Tianhong 2, the NPQ of Redchief, the qP of Gala, the NPQ of Starking, and the ETR of Yinv were significantly lower on 12–2 than on PYTC; whereas the qP of Starking, the NPQ of Luli and the qP and NPQ of Shoufu1 were all significantly higher on 12–2.

### Leaf antioxidant enzyme activities and MDA content

**Fig. 5:**
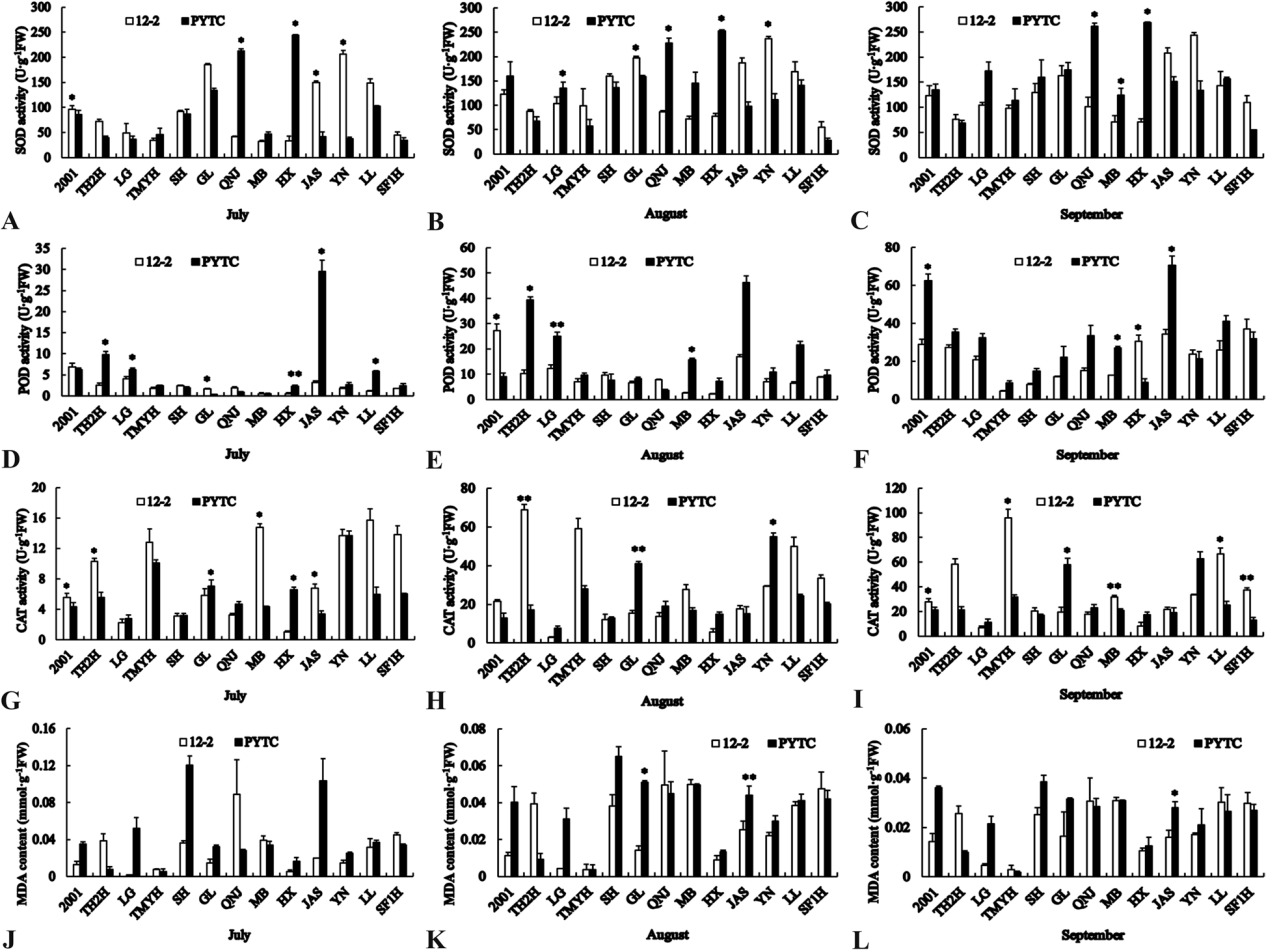
Leaf antioxidant enzyme activities and malondialdehyde contents of different scion–rootstock combinations. **A**–**C** SOD activity, **D**–**F** POD activity, **G**–**I** CAT activity, and **J**–**L** MDA content. *P < 0.05, **P < 0.01, Student’s *t*-test

In July, the activities of SOD and CAT in Fuji 2001, CAT in Tianhong 2, POD in Gala, CAT in USA8, SOD and CAT in Goldspur and SOD in Yinv were significantly higher on 12–2 than on PYTC (Fig. 5). However, the activities of POD in Tianhong 2, POD in Lvguang, CAT in Gala, SOD in Jonagold, all antioxidant enzymes in Starking, POD in Goldspur, and POD in Luli were significantly lower on 12–2. There were no significant differences in MDA content among the rootstock–scion combinations. In August, the activities of POD in Fuji 2001, CAT in Tianhong 2, SOD in Gala and SOD in Yinv were significantly higher on 12–2 than on PYTC. By contrast, the activities of POD in Tianhong 2, SOD and POD in Lvguang, CAT in Gala, SOD in Jonagold, POD in USA8, SOD in Starking, and CAT in Yinv were significantly lower on 12–2 than on PYTC. The MDA contents of Jonagold and Goldspur were also significantly lower on 12–2 than on PYTC in August. In September, the activities of SOD in Fuji 2001, CAT in Gala, SOD in Jonagold, SOD and POD in USA8, SOD in Starking and POD in Goldspur and the MDA content of Goldspur were all significantly lower on 12–2 than on PYTC. However, the activities of CAT in Fuji 2001, Tengmu No.1, USA8 and Shoufu1, and the activity of POD in Starking were significantly higher on 12–2.

### Principal component analysis

**Fig. 6:**
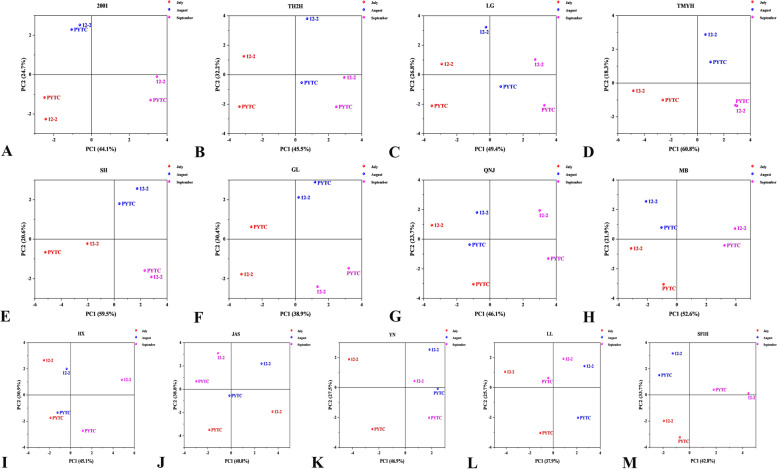
PCA analysis of different scion–rootstock combinations: **A** Fuji 2001, **B** Tianhong 2, **C** Lvguang, **D** Tengmu No.1, **E** Redchief, **F** Gala, **G** Jonagold, **H** USA8, **I** Starking, **J** Goldspur, **K** Yinv, **L** Luli, and **M** Shoufu1

The results by Origin software showed that Fuji 2001, Tengmu No.1, Redchief, Gala, USA8 and Shoufu1 had similar growth vigor on the two rootstocks (Fig. 6). And as shown in Table 3, Tianhong 2, Jonagold, Goldspur, Yinv and Luli were all characterized by four principal components, whereas Lvguang and Starking were characterized by three.

**Table 3:** Eigenvectors and percentages of accumulated contribution of principal components in different scion–rootstock combinations

	**TH2H**				**LG**			**QNJ**				**HX**		
indices	*F*_*1*_	*F*_*2*_	*F*_*3*_	*F*_*4*_	*F*_*1*_	*F*_*2*_	*F*_*3*_	*F*_*1*_	*F*_*2*_	*F*_*3*_	*F*_*4*_	*F*_*1*_	*F*_*2*_	*F*_*3*_
Height	0.499	0.689	-0.378	0.171	0.570	0.760	-0.310	0.652	0.653	0.195	0.314	0.778	0.601	0.173
Diameter	0.765	0.013	0.310	0.564	0.249	0.823	-0.505	0.340	0.924	-0.136	-0.073	0.695	0.695	0.111
SPAD	0.405	0.420	0.507	-0.635	0.339	0.698	-0.519	0.316	0.258	-0.786	-0.381	0.574	0.731	0.143
* P*_n_	-0.598	0.762	-0.133	0.012	-0.867	0.421	-0.048	-0.731	0.609	0.075	0.238	-0.677	0.631	0.206
* C*_i_	-0.608	0.549	0.566	0.059	-0.966	0.197	-0.015	-0.805	-0.221	-0.202	0.302	-0.587	0.381	-0.660
* G*_s_	0.138	0.878	-0.450	0.074	-0.156	0.808	0.538	-0.524	0.552	0.628	0.132	-0.535	0.667	0.261
* T*_r_	-0.626	0.456	-0.591	-0.217	-0.762	0.137	0.630	-0.862	0.131	0.479	-0.036	-0.861	0.302	0.284
ETR	0.962	-0.226	0.114	-0.065	0.438	0.391	0.787	0.888	0.169	0.413	-0.021	0.777	0.119	0.609
* Φ*_PSII_	0.788	-0.490	0.186	-0.196	0.969	-0.089	-0.224	0.800	0.266	-0.384	0.333	0.706	-0.250	-0.644
qP	-0.709	-0.528	0.188	0.386	0.867	-0.232	-0.414	0.537	0.696	-0.248	-0.265	0.939	-0.007	-0.329
NPQ	0.967	0.195	0.020	0.126	0.626	0.620	0.470	-0.172	0.482	0.679	-0.519	0.138	-0.192	0.938
* F*_v_/*F*_m_	0.956	0.034	-0.250	0.136	0.612	0.338	0.700	0.823	-0.313	0.229	-0.088	-0.977	-0.113	0.075
SOD	0.564	0.767	0.155	-0.248	0.892	-0.125	0.339	0.423	-0.773	0.440	0.076	-0.157	-0.978	0.132
POD	0.720	-0.474	-0.381	-0.326	0.910	-0.314	0.252	0.863	-0.018	0.019	0.498	0.943	0.027	-0.021
CAT	0.634	0.653	0.033	0.391	0.866	-0.470	0.083	0.772	0.145	0.562	0.179	0.305	-0.815	0.378
MDA	-0.075	0.891	0.447	-0.025	-0.304	-0.778	0.356	-0.765	0.417	-0.217	0.427	0.030	-0.857	0.040
Eigenvectors	7.280	5.153	1.902	1.355	7.908	4.283	3.174	7.369	3.797	2.795	1.343	7.211	4.939	2.631
Contribution rate (%)	45.500	32.203	11.885	8.469	49.424	26.771	19.835	46.058	23.728	17.472	8.391	45.069	30.872	16.445
Accumulate contribution rate (%)	45.500	77.703	89.588	98.057	49.424	76.195	96.030	46.058	69.786	87.258	95.649	45.069	75.941	92.386
	**JAS**				**YN**					**LL**				
indices	*F*_*1*_	*F*_*2*_	*F*_*3*_	*F*_*4*_	*F*_*1*_	*F*_*2*_	*F*_*3*_	*F*_*4*_		*F*_*1*_	*F*_*2*_	*F*_*3*_	*F*_*4*_	
Height	0.474	0.850	-0.004	-0.185	0.537	0.829	0.035	-0.093		0.752	0.583	-0.020	0.305	
Diameter	0.592	0.729	-0.303	0.119	-0.091	0.939	0.270	-0.169		-0.093	0.823	0.021	0.555	
SPAD	0.083	0.949	0.266	0.130	0.907	-0.287	0.249	0.058		-0.203	0.885	0.301	0.252	
* P*_n_	0.870	-0.130	0.375	0.287	0.572	0.602	-0.128	0.542		0.607	0.443	0.488	-0.290	
* C*_i_	0.669	-0.538	-0.508	0.065	-0.950	0.069	0.235	0.153		-0.852	-0.014	0.402	0.318	
* G*_s_	0.805	0.023	0.429	0.409	0.715	0.531	-0.443	0.092		0.794	0.052	0.564	-0.179	
* T*_r_	0.729	-0.372	0.502	0.266	0.153	0.382	-0.908	-0.074		0.466	-0.400	0.789	0.010	
ETR	-0.508	0.799	0.243	-0.118	0.543	-0.802	0.046	0.202		0.902	0.119	-0.181	-0.372	
* Φ*_PSII_	-0.961	0.110	-0.022	0.250	0.920	-0.056	0.148	-0.340		0.697	-0.219	-0.388	0.360	
qP	-0.850	0.446	0.070	0.087	0.693	-0.294	0.459	-0.449		0.578	-0.132	-0.581	0.540	
NPQ	0.224	0.470	0.546	-0.598	0.865	0.483	0.025	0.011		0.730	-0.208	0.034	0.156	
* F*_v_/*F*_m_	-0.765	-0.544	0.156	0.308	0.950	-0.119	-0.150	-0.222		0.795	-0.579	0.003	0.160	
SOD	0.300	0.884	-0.347	0.017	0.128	0.840	0.526	-0.003		0.280	0.793	0.346	-0.414	
POD	-0.852	0.193	0.148	0.353	0.620	-0.203	0.697	0.211		0.297	0.163	-0.692	-0.634	
CAT	-0.218	0.944	0.063	0.223	0.823	-0.249	0.068	0.488		0.571	0.720	-0.213	0.279	
MDA	-0.395	-0.731	0.343	-0.399	0.514	-0.403	-0.743	-0.103		0.472	-0.565	0.595	0.285	
Eigenvectors	6.525	6.211	1.648	1.259	7.497	4.397	2.805	1.074		6.072	4.110	2.952	2.023	
Contribution rate (%)	40.779	38.816	10.300	7.871	46.858	27.481	17.534	6.710		37.948	25.686	18.450	12.646	
Accumulate contribution rate (%)	40.779	79.595	89.896	97.767	46.858	74.338	91.873	98.583		37.948	63.634	82.084	94.730	

The contribution rates of the first four principal components in Tianhong 2 were 45.500%, 32.203%, 11.885%, and 8.469%, and their combined contribution rate was 98.057% (> 85.000%), indicating that the first four principal components represented 98.057% of the comprehensive information on all measured traits in the original data. The first principal component was determined by ETR, *Φ*_PSII_, NPQ, *F*_v_/*F*_m_ and POD; the second principal component by shoot height, *P*_n_, *G*_s_, *T*_r_, SOD and MDA; the third by SPAD and *C*_i_; and the fourth by qP, shoot diameter and CAT.

The contribution rates of the first three principal components in Lvguang were 49.424%, 26.771%, and 19.835%, and their combined contribution rate was 96.030%. The first principal component was determined by *Φ*_PSII_, qP, SOD, POD and CAT; the second by shoot height, shoot diameter, SPAD, *P*_n_, *C*_i_, *G*_s_ and NPQ; and the third by *T*_r_, ETR, *F*_v_/*F*_m_ and MDA.

The contribution rates of the first four principal components in Jonagold were 46.058%, 23.728%, 17.472%, and 8.391%, and their combined contribution rate was 95.649%. The first principal component was determined by ETR, *Φ*_PSII_, *F*_v_/*F*_m_ and POD; the second by shoot height, shoot diameter, SPAD, *P*_n_ and qP; the third by *G*_s_, *T*_r_, NPQ, SOD and CAT; and the fourth by *C*_i_ and MDA.

The contribution rates of the first three principal components in Starking were 45.069%, 30.872%, and 16.445%, and their combined contribution rate was 92.386%. The first principal component was determined by shoot height, *Φ*_PSII_, qP and POD; the second by shoot diameter, SPAD, *P*_n_, *C*_i_ and *G*_s_; and the third by *T*_r_, ETR, NPQ, *F*_v_/*F*_m_, SOD, CAT and MDA.

The contribution rates of the first four principal components in Goldspur were 40.779%, 38.816%, 10.300%, and 7.871%, and their combined contribution rate was 97.767%. The first principal component was determined by *P*_n_ and *C*_i_; the second by shoot height, shoot diameter, SPAD, ETR, qP, SOD and CAT; the third by *T*_r_, NPQ and MDA; and the fourth by *G*_s_, *Φ*_PSII_, *F*_v_/*F*_m_ and POD.

The contribution rates of the first four principal components in Yinv were 46.858%, 27.481%, 17.534%, and 6.710%, and their combined contribution rate was 98.583%. The first principal component was determined by SPAD, *G*_s_, ETR, *Φ*_PSII_, NPQ, *F*_v_/*F*_m_ and MDA; the second by shoot height, shoot diameter, *T*_r_ and SOD; the third by qP and POD; and the fourth by *P*_n_, *C*_i_ and CAT.

The contribution rates of the first four principal components in Luli were 37.948%, 25.686%, 18.450%, and 12.646%, and their combined contribution rate was 94.730%. The first principal component was determined by shoot height, ETR, *Φ*_PSII_, NPQ, *F*_v_/*F*_m_ and POD; the second by shoot diameter, SPAD, SOD and CAT; the third by *P*_n_, *C*_i_, *G*_s_, *T*_r_ and MDA; and the fourth by qP.

As shown in Table 4. Except in July, the score of Jonagold grafted onto PYTC was higher than that of Jonagold grafted onto 12–2. Among the seven scion–rootstock combinations tested in other months, the scores of scions grafted onto 12–2 were higher than those of the same scions grafted on PYTC. These results indicated that the growth of Tianhong 2, Lvguang, Jonagold, Starking, Goldspur, Yinv, and Luli was better when they were grafted onto 12–2 than when they were grafted onto PYTC.

**Table 4:** Scores and sorting of principal components in different scion–rootstock combinations

**Treatment**	***F***_***1***_	***F***_***2***_	***F***_***3***_	***F***_***4***_	**score**	**sorting**
Tianhong 2						
12–2 (July)	-3.109	1.254	1.630	-1.241	-0.922	5
PYTC (July)	-3.374	-2.163	-0.483	1.456	-2.166	6
12–2 (August)	0.704	3.802	-0.765	0.881	1.529	1
PYTC (August)	0.378	-0.538	-2.083	-1.365	-0.365	4
12–2 (September)	2.943	-0.180	1.202	0.603	1.475	2
PYTC (September)	2.459	-2.175	0.499	-0.335	0.450	3
**Treatment**	***F***_***1***_	***F***_***2***_	***F***_***3***_		**score**	**sorting**
Lvguang						
12–2 (July)	-2.912	0.728	-1.559		-1.554	5
PYTC (July)	-3.513	-2.110	-0.172		-2.335	6
12–2 (August)	-0.241	3.229	1.280		0.999	2
PYTC (August)	0.653	-0.805	2.806		0.664	4
12–2 (September)	2.732	1.031	-1.928		1.244	1
PYTC (September)	3.281	-2.073	-0.427		0.982	3
**Treatment**	***F***_***1***_	***F***_***2***_	***F***_***3***_	***F***_***4***_	**score**	**sorting**
Jonagold						
12–2 (July)	-3.508	0.948	-1.804	1.125	-1.612	6
PYTC (July)	-0.999	-3.034	-0.971	-1.184	-1.449	5
12–2 (August)	-0.785	1.806	1.309	-0.223	0.277	3
PYTC (August)	-1.231	-0.364	2.542	-0.214	-0.227	4
12–2 (September)	3.005	1.946	-1.254	-1.136	1.531	1
PYTC (September)	3.518	-1.302	0.178	1.631	1.480	2
**Treatment**	***F***_***1***_	***F***_***2***_	***F***_***3***_		**score**	**sorting**
Starking						
12–2 (July)	-2.500	2.657	-1.399		-0.536	4
PYTC (July)	-1.886	-1.740	-1.511		-1.635	6
12–2 (August)	-0.368	1.998	1.545		0.705	2
PYTC (August)	-1.225	-1.344	2.436		-0.566	5
12–2 (September)	4.826	1.161	-0.424		2.463	1
PYTC (September)	1.153	-2.732	-0.647		-0.430	3
**Treatment**	***F***_***1***_	***F***_***2***_	***F***_***3***_	***F***_***4***_	**score**	**sorting**
Goldspur						
12–2 (July)	3.518	-1.924	-1.536	0.529	0.571	3
PYTC (July)	-1.860	-3.492	0.309	-1.337	-2.188	6
12–2 (August)	2.586	2.198	1.253	-1.120	1.949	1
PYTC (August)	-0.150	-0.550	1.645	1.644	0.024	4
12–2 (September)	-1.127	3.092	-1.073	-0.198	0.615	2
PYTC (September)	-2.967	0.677	-0.598	0.482	-0.971	5
**Treatment**	***F***_***1***_	***F***_***2***_	***F***_***3***_	***F***_***4***_	**score**	**sorting**
Yinv						
12–2 (July)	-4.266	1.892	0.016	0.953	-1.413	5
PYTC (July)	-2.499	-2.774	-0.981	-1.144	-2.182	6
12–2 (August)	1.831	2.537	-0.449	-1.260	1.392	1
PYTC (August)	2.449	-0.081	-2.328	0.935	0.780	3
12–2 (September)	0.692	0.443	2.338	-0.269	0.838	2
PYTC (September)	1.793	-2.017	1.405	0.785	0.585	4
**Treatment**	***F***_***1***_	***F***_***2***_	***F***_***3***_	***F***_***4***_	**score**	**sorting**
Luli						
12–2 (July)	-4.094	1.051	1.620	0.011	-0.983	5
PYTC (July)	-1.101	-3.033	-0.746	1.351	-1.164	6
12–2 (August)	2.653	1.437	1.973	0.608	1.817	1
PYTC (August)	2.101	-2.018	0.842	-1.042	0.302	3
12–2 (September)	0.867	1.923	-2.136	1.326	0.597	2
PYTC (September)	-0.426	0.640	-1.553	-2.254	-0.569	4

## Discussion

In this experiment, although the performance of different scion–rootstock combinations varied among different months, the overall graft survival rate on 12–2 did not differ from that on PYTC, indicating that 12–2 has good potential as an apple rootstock. After grafting, symbionts of this process are gradually formed between the rootstock and scion, so that nutrients and water can be transported between rootstock and scion. When the rootstock and scion are incompatible, the rootstock cannot transfer nutrients to the scion and they instead accumulate at the interface, causing it to swell and exhibit the phenomenon of ‘big and small feet’. The plant is in a state of ‘starvation’ for a long time, its vigor weakens and it eventually dies. Therefore, compatibility can be judged by observing the shape of the grafted interface [23]. In this experiment, multiple grafted interfaces on 12–2 and PYTC healed well and showed strong compatibility. Most of the semi-compatible scion–rootstock combinations were ‘big at the bottom and small at the top’, nevertheless these scion–rootstock combinations did not affect the growth of the scion. And the small number of ‘big on the bottom and small on the top’ combinations, it does not affect negatively the quality of the plant in terms of productivity and fruit quality.

At present, there have been few studies on the mechanical strength of apple rootstocks. Previous research by our group on the mechanical strength of the four candidate apple rootstocks SD1, SD2, SD3, and SD4 showed that the compressive, tensile and torsional strength of the four rootstocks were not inferior to those of rootstocks commonly used in production, such as *Malus micromalus*, PYTC, M.26, and *Malus prunifolia* var. Ringo [38]. There have been fewer reports on the mechanical strength of the grafted interface. The fusion process of the grafted interface involves cell division and differentiation between scion and rootstock, formation of plasmodesmata, differentiation and connection of the cambium and reconstruction of xylem and vascular bundle tissue [19, 33]. If the rootstock and scion are incompatible, the grafted interface will normally form a callus, but the parenchyma-callus will not fuse, the xylem will be only partially connected and phloem-transport tissue will not form [37]. These changes in cell and cell wall structure produce significant differences in mechanical strength [8]. The results of the present experiment showed that the mechanical strength of apple scions grafted onto 12–2 was not weak and was sometimes even better than that of the apple scions grafted onto PYTC. The results of the mechanical strength test also showed that mechanical strength parameters should be considered in future breeding work to eliminate rootstocks with poor mechanical strength and improve the efficiency of rootstock breeding.

Studies have shown that plant shoots with high graft compatibility grow more strongly [37]. The scion–rootstock combination of *Malus halliana* Koehne/ ‘Yanfu 6’ performed better than *Malus hupehensis*/ ‘Yanfu 6’ and other combinations, with strong growth and high photosynthetic ability [45]. Preliminary analysis on the growth of new shoots showed that most of the scions grafted onto 12–2 were larger than on the controls PYTC. However, because of the different weights given to each parameter in the comprehensive evaluation, assessments based on individual parameters alone will provide different results for grafting affinity and additional parameters will be needed for further assessment [10].

Photosynthetic rate can be used to assess the potential for plant growth and stress resistance [13]. Studies have shown that differences in the effects of rootstock on the scion are manifested mainly in the leaves. Different rootstocks can cause significant differences in the chlorophyll content of scion leaves, which in turn affects leaf photosynthetic rate [24]. *P*_n_ has been shown to increase with increasing SPAD value [43], and similar results were observed in this experiment (for example, in July Fuji 2001). In July and September, an overall increase in *P*_n_ and decrease in *C*_i_ indicated that the photosynthetic rate was higher at these times. By contrast, *C*_i_ was higher in August, indicating that assimilation efficiency was lower at this time [45]. The trend in *T*_r_ was basically the same as that in *C*_i_. Scions on 12–2 tended to have a lower transpiration rate. The differences seen in August may have occurred because light was stronger during this month, 12–2 may have had higher drought resistance, and photosynthesis is a comprehensive physiological indicator that is affected by both internal and external forces [2]. The specific mechanism underlying these differences remain to be explored.

Differences in fluorescence parameters between scions grafted onto 12–2 and PYTC occurred mainly in qP and NPQ. There were fewer significant differences in *F*_v_/*F*_m_, *Φ*_PSII_, and ETR. *F*_v_/*F*_m_ is used as an indicator of photoinhibition or PSII damage [4]. Based on fluorescence changes in 12–2 in a pot experiment, a reduction in qP was taken to infer that the ETR was inhibited [27]. Photosynthetic activity is maintained by activating acclimation mechanisms. These may include an increase in energy dissipation capacity, which can be detected as an increase in NPQ without changes in *F*_v_/*F*_m_. When stress exceeds acclimation capacity, permanent photoinhibition occurs, and this can be detected as a lower *F*_v_/*F*_m_ [20]. Under ARD, a degree of photoinhibition occurred in some scion–rootstock combinations, suggesting that ARD had some effect on the chlorophyll fluorescence of scions on 12–2 and PYTC. However, the relatively constant *F*_v_/*F*_m_ indicated that scions on both 12–2 and PYTC had strong resistance to ARD and could maintain a high maximum quantum efficiency of PSII, thereby making better use of light energy [35].

Peroxidase activity is the core of the plant antioxidant defense system [12], and studies have shown that ARD can reduce SOD, POD and CAT activities in PYTC seedlings [40]. Here, antioxidant enzyme activities differed between the same scions planted on 12–2 and PYTC, and rootstock effects differed among SOD, POD and CAT. For example, in July, SOD activity was significantly lower on 12–2 than on PYTC for Starking. By contrast, SOD activity was significantly higher on 12–2 than on PYTC for Goldspur, but POD activity was significantly lower. These results suggest that different scion–rootstock combinations may differ in their ability to effectively scavenge reactive oxygen species in the rootstock itself under stress and may therefore sustain different degrees of damage to membrane structure and function [28]. Different varieties on the same rootstock also differ in their degree of membrane damage and their ability to effectively scavenge reactive oxygen species under stress [46]. Here, several scion–rootstock combinations had significant differences in MDA content, consistent with this viewpoint.

Principal component analysis is the most commonly used multi-factor comprehensive evaluation method. The amount of information carried by a principal component is positively correlated with its contribution [10]. Zhao et al. [45] evaluated the grafting compatibility of different scion–rootstock combinations of *Malus halliana* Koehne and PYTC by principal component analysis. The results showed that the *Malus halliana* Koehne/ ‘Yanfu 6’ combination was better than other scion–rootstock combinations in terms of photosynthesis, fluorescence characteristics and graft affinity, but no advantages were found in other parameters. In this experiment, although the same analysis method was used, different software tools had their own advantages and disadvantages. Origin (Fig. 6) could visualize the differences among different scion–rootstock combinations, but it could not accurately analyze the results. SPSS (Tables 3, 4) produced a detailed analysis, but its results were not displayed attractively. For the different scion–rootstock combinations, the number of principal components and the parameters included in each principal component analysis were different. Although the 16 parameters measured in this experiment have some independence and some correlation, the results show that different scion–rootstock combinations must be analyzed separately, and no individual parameter is suitable for an overall analysis that can be unified and generalized. This shows that the selection of evaluation factors and evaluation methods will also affect the results of a comprehensive evaluation of scion–rootstock combinations [45].

In addition, the difference in the physiological parameters between the two rootstocks in this experiment was obtained from the data of the first year of grafting. Differences in later fruit physiological parameters may have additional effects on the evaluation of grafting success, and these will be the focus of our next experiment.

## Conclusions

The grafting compatibility of 12–2 with 13 apple varieties studied under ARD conditions is better than that of the PYTC control, suggesting that 12–2 is an anti-ARD rootstock with promotion value in China.

## Data Availability

The datasets used and analysed during the current study are available from the corresponding author on reasonable request.

## Abbreviations

ARD: Apple replant disease
12–2: Apple rootstock superior line named 12–2
PYTC: *Malus hupehensis* Rehd.
2001: Fuji 2001
TH2H: Tianhong 2
LG: Lvguang
TMYH: Tengmu No.1
SH: Redchief
GL: Gala
QNJ: Jonagold
MB: USA8
HX: Starking
JAS: Goldspur
YN: Yinv
LL: Luli
SF1H: Shoufu1
*P*_n_: Net photosynthetic rate
*C*_i_: Intercellular CO_2_ concentration
*G*_s_: Stomatal conductance
*T*_r_: Transpiration rate
*F*_v_/*F*_m_: PSII original light energy conversion efficiency
*Φ*_PSII_: PSII actual photochemical efficiency
NPQ: Non-photochemical quenching coefficient
qP: Photochemical quenching coefficient
ETR: Electron transfer rate
MDA: Malondialdehyde
SOD: Superoxide dismutase
POD: Peroxidase
CAT: Catalase
